# Retraction Note: Quadratic multiple regression model and spectral relaxation approach for carreau nanofluid inclined magnetized dipole along stagnation point geometry

**DOI:** 10.1038/s41598-025-85406-3

**Published:** 2025-01-08

**Authors:** Sayed M. El Din, Adil Darvesh, Assad Ayub, Tanveer Sajid, Wasim Jamshed, Mohamed R. Eid, Syed M. Hussain, Manuel Sánchez-Chero, Sheda Méndez Ancca, Johana Milagritos Ramírez Cerna, Carmen Luisa Aquije Dapozzo

**Affiliations:** 1 Center of Research, Faculty of Engineering, Future University in Egypt, New Cairo, 11835 Egypt; 2 Department of Mathematics, Hazara University Mansehra, Mansehra, 21300 Pakistan; 3 Department of Mathematics, Government Post Graduate College Mansehra, Mansehra, 21300 Pakistan; 4 Department of Mathematics, Capital University of Science and Technology, Islamabad, 44000 Pakistan; 5 Department of Mathematics, Faculty of Science, New Valley University, Al-Kharga, Al-Wadi Al-Gadid 72511 Egypt; 6 Department of Mathematics, Faculty of Science, Northern Border University, Arar, 1321 Saudi Arabia; 7 Department of Mathematics, Faculty of Science, Islamic University of Madinah, Madinah, 42351 Saudi Arabia; 8 Universidad Nacional de Frontera, Sullana, Peru; 9 Universidad Nacional de Moquegua, Moquegua, Peru; 10 Universidad Alas Peruanas, Lima, Peru; 11 Universidad Nacional Tecnológica de Lima Sur, Lima, Peru

Retraction of: *Scientific Reports* 10.1038/s41598-022-22308-8, published online 15 October 2022

The Editors have retracted this Article as it contains several nonstandard phrases for which the authors have not been able to provide a suitable explanation. In addition the Article shows evidence of authorship irregularities, which the Authors were not able to satisfactorily explain. The Editors therefore no longer have confidence in the reliability of this Article and its metadata.

Authors Mohamed R. Eid and Syed M. Hussain disagree with this retraction. The other Authors have not responded to correspondence regarding this retraction.

